# Sequestrated Lumbar Disc Herniation Mimicking Spinal Neoplasm

**DOI:** 10.7759/cureus.18529

**Published:** 2021-10-06

**Authors:** Faisal Konbaz, Sami I Aleissa, Fahad Al Helal, Majed Abaalkhail, Waleed Alrogy, Abrar Bin Dohaim, Nasser Albishi

**Affiliations:** 1 Department of Orthopaedic Surgery, King Abdulaziz Medical City, Ministry of National Guard Health Affairs, Riyadh, SAU; 2 Department of Medicine, King Saud Bin Abdulaziz University for Health Sciences College of Medicine, Riyadh, SAU; 3 Department of Pathology, King Abdulaziz Medical City, Ministry of National Guard Health Affairs, Riyadh, SAU

**Keywords:** sequestrated, mimicking, neoplasm, herniated, lumbar disc

## Abstract

Sequestered disc fragments do not have indistinctive features and often share the clinical and radiological presentation as spinal neoplasms making their diagnosis and treatment a clinical challenge.

We report a rare case of sequestered lumbar disc fragment at the level of L2-L3 in a 70-year-old male who presented to the ER complaining of six years' history of low back pain with acute onset lower extremities weakness for six days, associated with right foot drop. He was admitted for tumor workup as the MRI showed diffuse bone high signal intensity throughout the spine with a soft tissue epidural mass at L2/3, causing severe compression on the cauda equina nerve roots. The patient underwent L2-L3 decompression and fixation, mass excision, multiple open biopsies. Soft tissue biopsy of the mass revealed fibrocartilaginous tissue consistent with the intervertebral disc, while the bone biopsy was diagnostic of acute leukemia. The patient was observed postoperatively with unremarkable complications. He did well with physiotherapy, and there was a remarkable improvement of his right lower extremity power reaching 4/5.

Our case presented a rare phenomenon in which sequestered disc fragments manifested clinically and radiologically as a spinal neoplasm. Vigilant history taking and physical examination are paramount; a physician should be watchful for any red flags that may warrant further investigation such as in our case.

## Introduction

Migration of herniated disc fragments is considered extremely rare given the contained anatomical barriers. These sequestered disc fragments do not have indistinctive features and often share the clinical and radiological presentation as spinal neoplasms making their diagnosis and treatment a clinical challenge [1,2]. In this paper, we review the literature and present a rare case of sequestered lumbar disc herniation mimicking spinal neoplasm resulting in acute foot drop and eventually led to diagnosing the patient with acute leukemia.

## Case presentation

**Figure 1:**
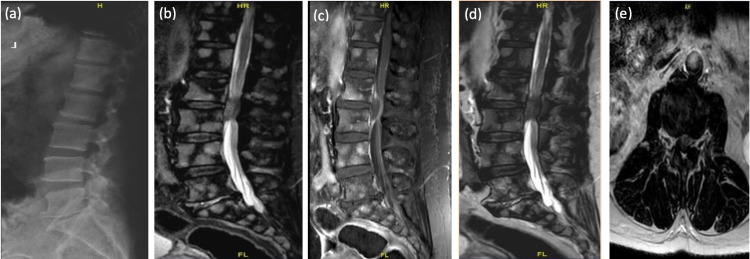
X-ray and lumbar MRI show preoperative changes. Preoperative x-ray of the lumbar spine showing degenerative changes without osseous abnormalities (a), sagittal STIR MRI (b), sagittal T1WI MRI without contrast (c) sagittal T2WI MRI without contrast (d), and axial T2WI MRI without contrast (e) demonstrating diffuse bone signals throughout the spine indicating neoplastic process, L2-L3 level anterior epidural soft tissue component with peripheral enhancement post-contrast causing significant compression of the thecal sac and cauda equina nerve roots.

**Figure 2:**
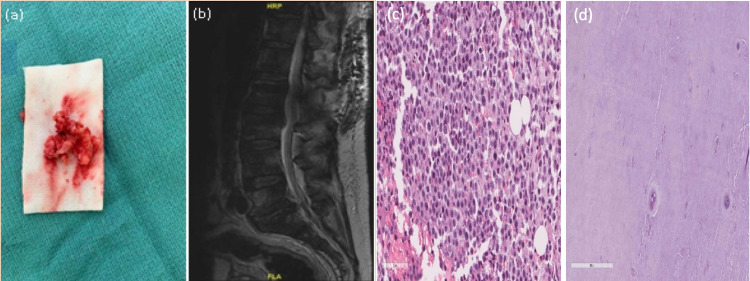
Tissue sample, postoperative lumbar MRI, and histopathology results. Intraoperative image of L2-L3 surgically removed disc fragment (a), postoperative sagittal T2WI MRI without contrast showing adequate decompression (b), a microscopic image of the bone biopsy showing marrow infiltration and high mitotic activity consistent with AML (c), and fibrocartilaginous tissue consistent with intervertebral disc material (d). AML - acute myeloid leukemia

This is a 70-year-old male, known to have hypertension, diabetes, benign prostatic hyperplasia, myocardial infarction on dual antiplatelets, presented to the ER complaining of six years' history of low back pain with acute onset lower extremities weakness for six days, associated with a new right foot drop. No reported history of traumas, or infections, no change in bowel and bladder habits. His examination revealed right-sided L2, L3, L4 power of 2/5, L5, S1 of 3/5. Sensation and reflexes were normal bilaterally. He was admitted for tumor workup as the MRI showed diffuse bone high signal intensity throughout the spine with a soft tissue epidural mass at L2/3 causing severe compression on the cauda equina nerve roots (Figures 1a-1e). The patient underwent L2-L3 decompression and fixation, mass excision, and open biopsies. Intra-operatively while excising the mass, grossly it looked like a sequestrated disc rather than a tumor. Bone biopsy revealed bone tissue with marrow infiltration suggestive of acute myeloid leukemia (AML). Soft tissue biopsy revealed fibrocartilaginous tissue consistent with intervertebral disc material (Figure 2). The patient was observed postoperatively with no complications. He did well with physiotherapy. Three months postoperatively he was able to ambulate and perform his daily activities. Physical exam showed that he had recovered normal motor function in the right foot. He is still on a treatment program with hematology for the AML. Follow-up MRI (Figures 2a-2d) showed adequate decompression was achieved at the L2/3 level.

## Discussion

A wide variety of non-neoplastic spinal diseases including sequestered intervertebral disc herniation may present as mass lesions making preoperative diagnosis and treatment a challenge for many surgeons. One of the early cases of sequestrated disc fragments was reported as protruded annulus fibrosus rotated posteriorly into the spinal canal [3]. The pathogenesis is not clearly understood yet. One hypothesis is anatomical derangement of the structures surrounding the lumbar spine such as age-related degenerative changes of lumbar discs, insufficiency of the supporting ligaments, or traumatic injuries that may facilitate sequestration of lumbar disc fragments [2,4].

We performed a PubMed and Google Scholar search of all reported studies of sequestrated lumbar disc herniation published to date. The following keywords were searched: Sequestered disc herniation, lumbar disc fragment, and spinal neoplasm. We identified 38 cases of lumbar disc fragments from 12 studies (Table 1). Only cases with a preoperative diagnosis of spinal tumors confined to the lumbar region were included. Analysis of the literature revealed that the majority of cases were males accounting for 61%. The mean age was 56.06 years. Symptoms range from low back pain, muscle weakness to cauda equina syndrome [2,5]. The most common presenting symptom was cauda equina syndrome representing 41% of all symptoms. Although any spinal level might be affected, sequestered disc herniation has a predilection for the upper levels of the lumbar spine. It has been investigated that congenital, post-traumatic, postoperative, or inflammatory adhesions, forming predominantly in the lumbar spine predispose it to more sequestration of disc fragments compared to other spinal levels [2,5]. Our literature review showed that the most common location of sequestrated lumbar disc herniation was at the level of L3-L4 accounting for 37% of all cases [6-15].

**Table 1:** Summary of all reported cases of sequestered lumbar disc herniation mimicking spinal tumors/neoplasms. LBP - low back pain, RP - radicular pain, CES - cauda equina syndrome, T1WI - T1-weighted image, T2WI - T2-weighted image, Hypo - hypointense, Hyper - hyperintense, Iso - isointensive, RE - rim enhancement, - - not reported.

Study	Mean Age	Gender	Symptoms	Level	T1W1	T2W1	CE	Preoperative Diagnosis
Eysel and Herbsthofer, 2001 [7]	41	2 M; 1 F	CES, LBP	2 L3–L4; 1 L4–L5	-	-	-	Spinal tumor
Nievas and Hoellerhage, 2009 [8]	59.5	2 M; 4 F	RP, LBP, CES	1 T12-L1 2 L1-L2; 2 L4-L5; 1 L5-S1	Iso; hypo; hyper	Iso; hypo; hyper	RE	Neoplasm; schwannoma; meningioma; epidural metastasis
Demirci and Er, 2010 [9]	53	F	LBP	L2-L3	Hypo	Hypo	RE	Spinal tumor
Akhaddar et al., 2011 [4]	52	F	RP, LBP, CES	L2–L3	-	-	-	Epidural abscess/neoplasm
Sengoz et al., 2011 [10]	47.9	6 M; 2 F	CES, RP	6 L3–L4; 2 L4–L5	-	-	RE	Spinal tumor
Biasi et al., 2013 [11]	60	F	LBP	L4-L5	Hypo	Hyper	RE	Spinal tumor
Li et al., 2015 [12]	48	M	CES	L5-S1	Hypo	Hypo	RE	Spinal tumor
Ajayi et al., 2016 [13]	65	F	CES	L3-L4	Hypo	Hyper	RE	Spinal tumor
Turan et al., 2017 [14]	49.6	7 M; 2 F	CES, LBP, RP	1 L2–L3; 4 L3–L4; 3 L4–L5; 1 L5–S1	Iso; hypo	Iso; hyper	RE	Disc herniation, spinal tumor
Kim et al., 2018 [15]	76	M	RP	L2-L3	Iso	Hyper	RE	Epidural neoplasm
Afonso et al., 2018 [1]	54.5	2 M	CES, LBP, RP	L3-L4; L4- L5	Iso	Hypo; hyper	RE	Spinal tumor; meningioma
Rai et al., 2020 [2]	52.3	1 M; 2 F	CES	2 L2-L3; 1 L5-S1	Hypo	Hypo	RE	Extradural tumor, epidural mass
Present study	70	M	LBP	L2-L3	-	-	RE	Spinal neoplasm

Contrast injection is used to differentiate between avascular disc fragments from other spinal neoplasms with the presence of rim-like peripheral enhancement. This finding is suggestive more of an inflammatory and non-neoplastic process [6]. Other conditions such as synovial cysts, epidural abscesses, or hematomas might share similar MR intensity, and rim enhancement [1,2,5,8]. The atypical presentation of sequestrated disc fragments along with the past medical history of malignancy such as in our case can lead to the consideration of spinal neoplasms as one of the top differential diagnoses. As a result, unnecessary investigations for malignancy might delay diagnosis and treatment, add more cost, and most importantly may result in considerable anxiety for patients misdiagnosed with spinal neoplasms.

## Conclusions

Our case presented a rare phenomenon in which sequestered disc fragments manifested clinically and radiologically as a spinal neoplasm. Vigilant history taking and physical examination are paramount; a physician should be watchful for any red flags that may warrant further investigation such as in our case. Sequestrated disc fragments should be included in the differential diagnosis of patients presenting with low back pain, radiculopathy, or cauda equina syndrome.
